# Effect of Commercial and Autochthonous Bioprotective Cultures for Controlling *Listeria monocytogenes* Contamination of Pecorino Sardo Dolce PDO Cheese

**DOI:** 10.3390/foods12203797

**Published:** 2023-10-16

**Authors:** Maria Pina Meloni, Francesca Piras, Giuliana Siddi, Mattia Migoni, Daniela Cabras, Mario Cuccu, Gavino Nieddu, Olivia McAuliffe, Enrico Pietro Luigi De Santis, Christian Scarano

**Affiliations:** 1 Department of Veterinary Medicine, University of Sassari, Via Vienna, 2, 07100 Sassari, Italy; mpmeloni@uniss.it (M.P.M.); g.siddi1@phd.uniss.it (G.S.); mattiamigoni35@gmail.com (M.M.); d.cabras1991@libero.it (D.C.); mrcuccu@uniss.it (M.C.); desantis@uniss.it (E.P.L.D.S.); scarano@uniss.it (C.S.); 2 Cooperativa Allevatori Ovini Formaggi Soc. Coop. Agricola, Loc. “Perda Lada” Fenosu, 09170 Oristano, Italy; gavino.nieddu@caoformaggi.it; 3 Teagasc Food Research Centre, Moorepark, Fermoy, P61 C996 Co., P61 C996 Cork, Ireland; olivia.mcauliffe@teagasc.ie

**Keywords:** foodborne pathogens, food biopreservation, lactic acid bacteria, *Lactiplantibacillus plantarum*, *Lactobacillus delbruekii* sups. *sunkii*, challenge test, protected designation of origin cheese

## Abstract

The composition and physicochemical characteristics of short-aged Pecorino Sardo PDO (Protected Designation of Origin) cheese makes it permissive to *Listeria monocytogenes* growth. The PDO product specification stipulates that this cheese is produced with whole sheep’s milk inoculated with cultures from the area of origin. Therefore, the use of bioprotective cultures for the inhibition of pathogens in PDO cheeses is allowed only if autochthonous microorganisms are used. Furthermore, bioprotective cultures are generally used on the cheese surface to prevent the outgrowth of *L. monocytogenes*, the application of which can be time-consuming and require specialist technical knowledge. In this study, we examine the direct addition of bioprotective cultures to the cheese vat and compare the activity of a commercial bioprotective culture (*Lactiplantibacillus plantarum*) and an autochthonous lactic acid bacterium with bioprotective properties (*Lactobacillus delbruekii* sups*. sunkii*), for the inhibition of *L. monocytogenes* in Pecorino Sardo PDO cheese. Three types of Pecorino Sardo PDO cheese were made with bioprotective cultures added directly to the cheese milk along with the starter inoculum: PSA, with the commercial bioprotective culture; PSB, with the autochthonous bioprotective culture; and a CTRL cheese with no bioprotective culture. A challenge test was performed on each of these cheeses by artificially contaminating the cheese surface with *L. monocytogenes* (2 Log10 CFU/g). Three batches of each cheese type were analyzed to enumerate mesophilic and thermophilic lactic acid bacteria and to investigate the growth potential of *L. monocytogenes* during manufacturing, at the end of ripening, at the end of shelf-life, and after 180 days from cheese production. Both bioprotective cultures tested in this study showed inhibitory action against the pathogen with 0.3–1.8 Log10 CFU/g (colony-forming unit per gram) reduction levels. The autochthonous organism, *L. sunkii*, was as effective as the commercially supplied culture, and the addition of the bioprotective cultures to the cheese-making procedure offered protection against *L. monocytogenes*. The direct addition of bioprotective cultures to the making procedure of Pecorino Sardo PDO cheese is a potentially innovative strategy to improve the safety of this product.

## 1. Introduction

The dairy sector is economically relevant in Italy. Sardinia produces 2% of Italian cow’s milk and 68% of Italian sheep’s milk [1]. Considering these data, Sardinia is classified as the primary region within the European Union for sheep milk production (13% of the total) as well as being the leading region for sheep cheese exportation [2]. The European Union has awarded the PDO label to over 180 cheeses and 3 of these are made in Sardinia: “Pecorino Romano”, “Fiore Sardo”, and “Pecorino Sardo” [3]. In Sardinia in 2020, 309.631 tons of sheep’s milk were collected, 176.776 tons of which were used to produce Pecorino Romano, 13.403 tons to produce Pecorino Sardo, and almost 5.896 tons to produce Fiore Sardo [1]. Pecorino Sardo is a half-cooked paste cheese produced with ewe’s milk. According to the ripening time, there are two types of Pecorino Sardo cheese: “dolce”, ripened for 20–60 days, and “maturo”, ripened for >60 days [4]. The production specification of Pecorino Sardo PDO stipulates that “the whole sheep milk is inoculated only with bacteria from the origin area” [5].

*L. monocytogenes* is a Gram-positive bacterium, responsible for human listeriosis, a serious foodborne infection. The organism is widespread in the environment [6] and it can survive in a wide range of environmental conditions, posing a risk of food contamination, and consequently a risk for human health. It is a particular issue in ready-to-eat (RTE) foods, i.e., foods which do not undergo any heat treatment before consumption, such as Pecorino Sardo Dolce PDO. In accordance with Regulation (EC) No 2073/2005 [7], if a food does not comply with the microbiological criteria about the *L. monocytogenes* presence, the food business operator (FBO) shall apply measures to recall/withdraw products. This bacterium has been recurrently isolated on food industry surfaces despite regular cleaning and disinfection practices. The capacity of *L. monocytogenes* to resist cleaning procedures contributes to making it persistent and very difficult to eradicate. Cheese portioning and packaging areas are the most affected by *L. monocytogenes* contamination and, therefore, represent a large risk of cross-contamination for foods [8]. Therefore, to ensure the safety of consumers, it is necessary to implement measures to control the occurrence and persistence of this pathogen in Pecorino Sardo PDO processing. Proper hygiene and disinfection practices are, therefore, essential to prevent the post-process contamination of cheeses [9].

The use of lactic acid bacteria (LAB) as bioprotective cultures has been well documented. Their biopreservative properties are related to their ability to produce several antimicrobial compounds, such as bacteriocins, organic acids, and hydrogen peroxide, and their inclusion in product manufacture could provide an additional food safety guarantee [10]. This property and the increased consumer interest in clean-label foods make LAB particularly advantageous for the food industry, offering natural alternatives to chemical additives [10,11]. In this study, two bioprotective cultures, one commercial and one autochthonous lactic acid bacterium, were evaluated for their ability to inhibit *L. monocytogenes* in Pecorino Sardo dolce PDO during storage. The bioprotective cultures were inoculated in milk during the cheesemaking process. The chosen commercial culture consisted of a *Lactiplantibacillus plantarum* strain bacteriocin producer, whereas the autochthonous bioprotective bacterium was isolated from Sardinian raw sheep milk and it was identified as a *Lactobacillus delbruekii* sups*. sunkii*. As a medium for the inoculation of these microorganisms in milk, “scotta” was chosen. “Scotta” is the residual whey from Ricotta cheese production and it is inoculated with the starter cultures for the production of Pecorino Romano PDO and Pecorino Sardo PDO cheeses, forming the “*scotta-innesto*” [12]. In a previous study [13], a modified *scotta-innesto* was prepared by inoculating *L. plantarum* and *L. sunkii* that showed in vitro antilisterial activity and excellent growth ability in this medium. These results showed that the “scotta” could be an excellent medium for inoculation of bioprotective cultures in milk during the cheese production process and, therefore, it was used in this study. A challenge test was conducted on cheese inoculated with these bioprotective cultures to investigate their antilisterial activity and *L. monocytogenes* growth potential during Pecorino Sardo shelf-life at different storage temperatures. For experimental contamination, *L. monocytogenes* field strains isolated from Sardinian cheesemaking plants were used to perform a targeted study of pathogenic strains easily detectable in cheese-processing environments.

## 2. Materials and Methods

In order to obtain cheese samples for experimentation, two bioprotective cultures (one commercial and one autochthonous) were introduced into the production process of Pecorino Sardo Dolce PDO cheese at a cheesemaking partner of this project, resulting in PSA cheese, with commercial culture, and PSB cheese, with autochthonous culture. Additionally, control cheeses (CTRL) were produced without the utilization of bioprotective cultures.

### 2.1. Preparation of Inoculum of Bioprotective Cultures

In order to inoculate the bioprotective cultures during the cheesemaking process, a *scotta-innesto* for each experimental production was prepared.

*Scotta-innesto* was produced using the following procedure:

1. PSA *scotta-innesto*: Following the manufacturer’s instructions, 4.5 g of lyophilized commercial protective culture (Sacco-Lyofast LPAL, Sacco System, Cadorago, Italy) was inoculated into 30 L of “*scotta*” and incubated at 37 °C for 17 h before use to reach a concentration of 10^6^ CFU/mL.
2. PSB *scotta-innesto*: One liter of a stock culture of *Lactobacillus delbrueckii* subsp. *sunkii* was added to 30 L of “*scotta*” and incubated at 45 °C for 17 h before use to reach a concentration of 10^6^ CFU/mL.

**Figure 1:**
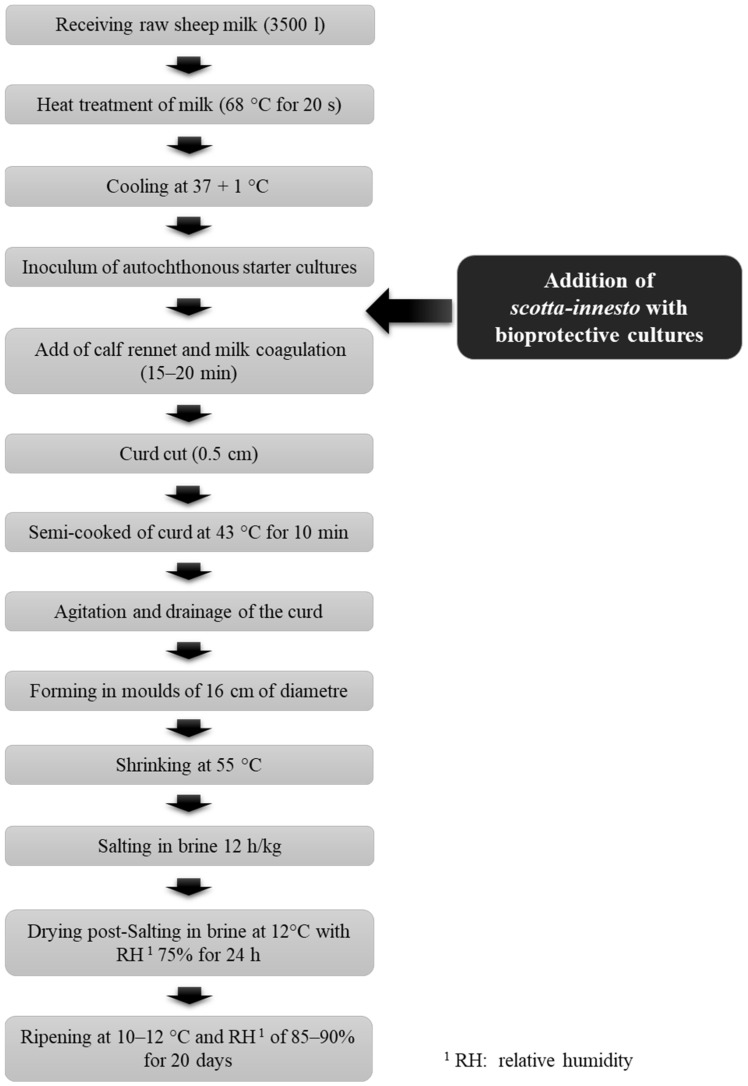
Flow chart of the Pecorino Sardo Dolce PDO production.

The PSA and PSB *scotta-innesto* were added to the milk in the cheese vats immediately after the inoculation of the starters and before the coagulation phase. For the control (CTRL) production, the process described in Figure 1 (and in Section 2.2) was followed without the addition of *scotta-innesto*.

### 2.2. Production Process of Pecorino Sardo PDO Cheese

The milk was treated at 68 °C for 20 s, then cooled to 37 + 1 °C and inoculated with autochthonous starter cultures and coagulated with commercial calf rennet. Milk coagulation occurred in 15–20 min and the curd was cut into grains of about 0.5 cm and cooked to 43 °C for 10 min. The curd was poured into moulds and transferred to a warm room at a temperature of 55 °C. Once a pH of 5.2 was reached, the cheeses were salted in brine for 24 h and then ripened at 10–12 °C and relative humidity of 85–90%. Afterwards, a ripening time of 20 days was applied to obtain Pecorino Sardo “dolce”.

### 2.3. Experimental Design

At a cheesemaking plant located in Sardinia, three batches of cheese were produced on three separate days, in order to ensure the reproducibility of the results.

For each batch, three cheese vats with 3500 L of milk were used to produce:

1. PSA cheese: Pecorino Sardo Dolce inoculated with a *scotta-innesto* containing a freeze-dried commercial protective culture (PC) consisted of a selected *Lactiplantibacillus plantarum* bacteriocin producer;
2. PSB cheese: Pecorino Sardo Dolce inoculated with a *scotta-innesto* containing a strain of *Lactobacillus delbrueckii* subsp. *sunkii* (LS) isolated from Sardinian raw sheep’s milk;
3. CTRL cheese: Pecorino Sardo Dolce without *scotta-innesto*. It was produced respecting the traditional production process (Figure 1) and it was used as a control.

For all experimental productions, the manufacturing procedure of Pecorino Sardo PDO followed the PDO indications [5] (Figure 1). The only change made was the addition of *scotta-innesto* for PSA and PSB.

### 2.4. Preparation of the L. monocytogenes Inoculum

A total of five strains of *L. monocytogenes* were used to create the pathogen inoculum, consisting of three reference strains and two field strains. The selection of these strains aimed to include the most common serotypes of *L. monocytogenes* and resident strains, ensuring a representative pool for experimental contamination [14,15,16]. Their characteristics are described as follows:

1. *L. monocytogenes* ATCC19111, serotype 1/2a (reference strain);
2. *L. monocytogenes* NCTC10887, serotype 1/2b (reference strain);
3. *L. monocytogenes* ATCC19115, serotype 4b (reference strain);
4. *L. monocytogenes* LM837, serotype 1/2b (isolated from a floor drain in a cheesemaking plant);
5. *L. monocytogenes* LM843, serotype 1/2c (isolated from a salted ricotta produced in a partner dairy industry).

According to the “EURL Lm Technical guidance document on challenge tests and durability studies for assessing shelf-life of ready-to-eat foods related to *L. monocytogenes*” (version 4 of 1 July 2021), each strain was previously subcultured and incubated at 37 °C for 16 h to ensure reaching the stationary phase. To acclimate the cells to the expected environmental conditions for the challenge test, the bacterial suspensions were stored at +4 °C for 7 days. Once it was confirmed that the microbial concentration was equal to 10^9^ CFU/mL, the 5 cultures of *L. monocytogenes* were mixed together in equal volumes and the total volume was diluted. The pool preparation was standardized through preliminary experimentation: the levels of *L. monocytogenes* for each inoculum were 3 Log10 CFU/mL in order to achieve a final concentration of 100 CFU/g on cheese.

### 2.5. Experimental Contamination of Pecorino Sardo PDO Cheese

A challenge test on portioned PSA, PSB, and CTRL cheese was set up in order to test and compare the ability of commercial and autochthonous bioprotective cultures to inhibit *L. monocytogenes* in Pecorino Sardo Dolce PDO. For the challenge test design, the “EURL Lm Technical guidance document on challenge tests and durability studies for assessing shelf-life of ready-to-eat foods related to *L. monocytogenes*” (version 4 of 1 July 2021) was used as reference [17].

At the end of the ripening period (20 days), the three types of cheese (PSA, PSB, and CTRL) were cut into 300 g pieces using sterile knives. They were then contaminated with the prepared inoculum of *L. monocytogenes,* that had been previously adapted to the storage temperatures of the cheese (+4 °C) in order to simulate contamination during portioning. A sterile spatula was used to contaminate the cheese surfaces, with the inoculum spread across the two sides of each portion. All of these steps were conducted inside a laminar flow cabinet to ensure the sterility and safety of the process. Additionally, for each type of cheese (PSA, PSB, and CTRL), six pieces of cheese per batch were served as “blank” controls (negative controls), that is, not contaminated with the *L. monocytogenes* pool but inoculated with sterile 0.85% NaCl. Sampling activities were then conducted to assess the microbiological and physicochemical parameters of PSA, PSB, and CTRL throughout their shelf-life [18,19]. The contaminated and control samples were vacuum-packed and stored at two different temperatures: +4 °C, to simulate recommended storage conditions, and +10 °C, to simulate thermal abuse.

### 2.6. Sampling, Microbiological Analysis, and Evaluation of Physicochemical Parameters

#### 2.6.1. Sample Collection

PSA, PSB, and CTRL were sampled at the following analysis times:

- During cheesemaking, to verify the absence of *L. monocytogenes* in the cheese before experimental contamination (T_0_)
  - after breaking the curd;
  - after curd shrinking;
- On the day after the experimental contamination with *L. monocytogenes* (T_21_);
- Ninety days later (T_90_) which corresponds to the shelf-life of the product;
- One hundred and eighty days after production (T_180_) which corresponds to twice the duration of the shelf-life.

Samples were also taken at the above time points for the enumeration of mesophilic and thermophilic lactic acid bacteria [20], for the detection and enumeration of *L. monocytogenes* in compliance with the ISO 11290-1: 2017 [21] and ISO 11290-2: 2017 [22].

#### 2.6.2. Enumeration of Mesophilic and Thermophilic Lactic Acid Bacteria

To enumerate mesophilic and thermophilic LAB, 10 g of each sample was homogenized in a stomacher for one minute with 90 mL of 0.85% NaCl. The homogenized samples were then serially diluted (1:10). Subsequently, 1 mL of each dilution was plated on Man Rogosa and Sharpe (MRS) agar (pH 5.5, adjusted with 0.5 N HCl) (MRS, Biolife, Milan, Italy). The agar plates were incubated both at 30 °C and 45 °C under anaerobic conditions for 48–72 h.

#### 2.6.3. Detection of *Listeria monocytogenes*

To detect and enumerate *L. monocytogenes*, a 10 g sample was enriched in a 1:10 ratio in half-Fraser broth (Biolife, Milan, Italy) and incubated at 30 °C for 25 ± 1 h. Then, 0.1 mL of the initial enrichment culture was inoculated onto plates of Agar Listeria acc. to Ottaviani and Agosti (ALOA, Biolife, Milan, Italy) and incubated at 37 °C for 24 ± 2 h. Additionally, 0.1 mL of the half-Fraser broth was transferred to a tube containing 10 mL of Fraser broth (Biolife, Milan, Italy) and incubated at 37 °C for 24 ± 2 h. From the second enrichment, 0.1 mL was plated on ALOA (Biolife, Milan, Italy), and after incubation at 37 °C for 24 ± 2 h, typical colonies of *L. monocytogenes* were assessed. For enumerating *L. monocytogenes* and *Listeria* spp., 0.1 mL of the half-Fraser broth suspension was plated on a 90 mm ALOA plate (Biolife, Milan, Italy). After incubating at 37 °C for 24 ± 2 h, colonies of *L. monocytogenes* and *Listeria* spp. were counted. Three to five colonies with the characteristic appearance of *L. monocytogenes* (blue-green color surrounded by an opaque halo) were isolated from each positive sample. All isolates were stored at −80 °C in BHI broth (Brain Heart Infusion, Biolife, Milan, Italy) containing glycerol (15% *v*/*v*) for further analysis.

#### 2.6.4. Physicochemical Analysis

For each sample, the pH was measured using a pH meter (WTW 3110 profiline, Willis Towers Watson, London, UK), and the water activity (a_w_) was evaluated using the AQUALAB 4TE (METER Group, Inc., Pullman, WA, USA).

All tests were carried out in triplicate: for each sampling time, three samples of each cheese type were tested. The data obtained were compared using statistical analysis of variance (ANOVA, ANalysis of VAriance).

## 3. Results

Table 1 shows the results (mean levels + standard deviation Log10 CFU/mL) of the LAB enumeration in the samples of *scotta-innesto* PSA and *scotta-innesto* PSB, evaluated 17 h after inoculation.

Table 2 shows the results of the pH and a_w_ evaluation of the PSA, PSB, and CTRL cheeses. In Table 3, the results of mesophilic and thermophilic LAB enumeration (mean levels + standard deviation Log10 CFU/mL), and the qualitative and quantitative analysis of *L. monocytogenes* related to the three production batches during the challenge test are shown.

There were no significant differences (*p* > 0.05) in a_w_ mean values between inoculated (PSA and PSB) and non-inoculated (CTRL) samples stored at +4 °C and at +10 °C during the entire shelf-life. As expected, the a_w_ mean levels of PSA stored at +4 °C displayed a significant decrease (*p* < 0.01) at the following intervals: T_0_–T_90_, T_0_–T_180_, T_21_–T_90_, and T_21_–T_180_. On the other hand, for PSA samples stored at +10 °C, there was a significative decrease (*p* < 0.05) of a_w_ values during all sampling times (T_0_, T_21_, T_90_, and T_180_). The a_w_ values of PSB stored at +4 °C showed a significant decrease (*p* < 0.05) only at the end of the experiment (T_180_); no significant differences (*p* > 0.05) in a_w_ values for PSB stored at +10 °C between T_0_ and T_21_ were found, but a significative decrease (*p* < 0.05) between T_21_ and T_180_ emerged.

As expected, the pH trend of all samples showed a significant decrease (*p* < 0.01) between T_0_ and all following sampling times (T_21_, T_90_, and T_180_). No significant differences (*p* < 0.01) in pH were found between T_21_ and T_90_, between T_21_ and T_180_, or between T_90_ and T_180_ either for samples stored at +4 °C or for those stored at +10 °C. The samples of PSA and PSB (inoculated with bioprotective cultures) stored at +4 °C and +10 °C showed significantly higher (*p* < 0.01) pH values with respect to CTRL at T_90_; at the same sampling time, there were no significant differences (*p* < 0.01) between PSA and PSB at +4 °C and +10 °C. In relation to the storage of products at the correct refrigeration temperature (+4 °C) and at conditions of thermal abuse (+10 °C), no significant differences were observed (*p* > 0.05) for pH and a_w_ mean values. Therefore, the storage temperature did not affect the variations of these parameters.

At the end of the shelf-life, the minimum pH values were 4.8 ± 0.1 both for the samples stored at +4 °C and for those stored at +10 °C. The pH values slightly decreased up to 4.7 ± 0.1 at T_180_.

As for the a_w_ parameter, the minimum values found were 0.966 ± 0.01 at the end of the shelf-life (T_90_). A relevant decrease at T_180_ was noted, with some samples having a_w_ mean values of 0.925 ± 0.04.

The mean levels (Log10 UFC/g ± standard deviation) of thermophilic lactic microorganisms during storage at +4 °C showed a significant increase between T_0_ and T_21_ (*p* < 0.01) and a significant decrease (*p* < 0.01) of 0.3 Log10 at the end of shelf-life (T_90_) and of 0.8 Log10 beyond shelf-life (T_180_). For samples stored at +10 °C, the mean levels of thermophilic LAB were comparable to the samples stored at +4 °C. Considering the sampling times individually, there were no significant differences (*p* > 0.05) in the thermophilic LAB count between PSA, PSB, and CTRL samples stored at +4 °C. For the same samples stored at thermal abuse conditions, the thermophilic LAB mean levels were significative higher (*p* < 0.05) in the PSA and PSB samples at T_180_.

As for the mesophilic LAB trend, a significant increase (*p* < 0.01) was observed between T_0_ (7.2 ± 0.5) and T_21_ (8.0 ± 0.1). Afterward, the mean values decreased significantly (*p* < 0.01), reaching values similar to those detected at T_0_ (7.3 ± 0.5). At the end of the experiment (T_180_), a slight decrease was observed. The same trend occurred for samples stored at +10 °C.

In relation to the mesophilic LAB mean levels in inoculated samples, significative differences (*p* > 0.05) were observed only at T_90_ and T_180_ between the PSA and PSB samples stored at +4 °C. In this case, as expected, the mesophilic LAB counts in PSA were higher if compared to PSB. In samples stored at +10 °C, the mesophilic LAB mean values were significantly higher (*p* > 0.01) in PSA than in CTRL samples at T_90_ and T_180_; in the latter sampling time, the same evidence emerged for PSB samples.

No significant differences (*p* > 0.05) were found after comparison of the mean levels of mesophilic and thermophilic LAB related to the sampling times and to the storage temperatures used during the experiments.

As expected, *L. monocytogenes* was not detected at T_0_ using either qualitative or quantitative methods. The quantitative analysis showed levels between 0.3 and 1.8 Log10 UFC/g at T_21_ and the absence of *L. monocytogenes* at T_90_ and T_180_. The temperature did not affect the pathogen growth ability.

Overall, the two bioprotective cultures were effective against *L. monocytogenes*.

## 4. Discussion

In Italy, dairy products are economically important at the national level, and Sardinia in particular is a leading region in the export of sheep’s milk cheese, with a gross added value of approximately 320 million euros per year, which makes it the main region of the European Union for sheep’s milk production representing 13% of the total [2]. Pecorino Sardo Dolce PDO belongs to the category of ready-to-eat foods and, therefore, does not undergo further heat treatment before consumption. If a food does not fulfill the microbiological criteria for *L. monocytogenes* as defined by Regulation (EC) No 2073/2005 [7], the FBO shall apply product withdrawal/recall measures. Such a withdrawal or recall can be very damaging to both the reputation and economic stability of the company.

According to Regulation (EC) No 2073/2005 [7], the FBO must carry out studies on the shelf-life of RTE food in order to ensure that the limits for food safety criteria are complied with throughout its shelf-life. For this purpose, microbiological challenge testing can be used. Through the artificial contamination of food with a pathogen, it is possible to simulate and study the behavior of the pathogen during the product’s shelf-life [23].

Based on these considerations, a challenge test was designed and conducted on two types of Pecorino Sardo Dolce PDO (PSA and PSB) produced using a “*scotta-innesto*” inoculated, respectively, with *Lactiplantibacillus plantarum* (commercial bioprotective culture) and *Lactobacillus delbrueckii* subsp. *sunkii* (autochthonous bioprotective culture). In accordance with the “EURL Lm Technical guidance document on challenge tests and durability studies for assessing shelf-life of ready-to-eat foods related to *L. monocytogenes*” (version 4 of 1 July 2021) [14], the targeted level of contamination chosen was 100 CFU/g. This target level refers to what is defined in the Regulation (EC) No 2073/2005 [7], which specifies that manufacturer shall be able to demonstrate, to the satisfaction of the competent authority, that the product will not exceed the limit of 100 CFU/g throughout its shelf-life.

Although commercial bioprotective cultures are considered safe and easy to use [13], the *Lactobacillus delbrueckii* subsp. *sunkii* strain was selected to comply with the specification of Pecorino Sardo PDO which requires that the milk must be inoculated exclusively with natural and autochthonous cultures [24,25]. An additional type of Pecorino Sardo Dolce PDO was produced, respecting the traditional production process (without the addition of “*scotta-innesto*”), that was used as the experimental control (CTRL).

The choice to use *L. plantarum* and *L. sunkii* as bioprotective cultures was due to previous in vitro studies that had demonstrated their effectiveness in counteracting the growth of *L. monocytogenes* [13]. The positive results obtained by in vitro tests had to be confirmed by in vivo experiments on the cheese matrix. It is essential to understand the behavior of the two bioprotective bacteria when they are inoculated into the milk during cheese production and, specifically, if they maintain their antilisterial activity throughout the shelf-life of the product.

The results of the challenge test conducted on PSA, PSB, and CTRL products showed that *L. monocytogenes* is able to survive both in refrigeration conditions (+4 °C) and in conditions of thermal abuse (+10 °C). Starting from the same experimental contamination levels (100 CFU/g), the mean counts of *L. monocytogenes* showed no significant differences in relation to storage temperatures (*p* > 0.05).

Through the application of a quantitative analysis for *L. monocytogenes* determination, mean pathogen levels of 0.3–1.8 Log10 UFC/g at T_21_ (on the day after experimental contamination) were found, whereas the pathogen was absent at T_90_ (at the end of shelf-life) and 90 days after the end of shelf-life (T_180_). The mean levels of *L. monocytogenes* were significantly higher (*p* < 0.05) at T_21_ than at the end of shelf-life (T_90_).

These results are similar to those found in similar works: two recent studies on the effectiveness of LAB against *L. monocytogenes* in cheese [16,26] showed that the use of LAB with antilisterial activity determined the reduction of the pathogen from 2 to 3 Log10 UFC/g in the first 15–20 days after inoculation.

The results of the quantitative analysis showed the absence of *L. monocytogenes* at T_90_. The only positive results at T_90_ concerned the qualitative analysis, and 43.9% of these results were related to CTRL samples (without the use of bioprotective culture); at T_180_, no *L. monocytogenes* was detected in PSA and PSB samples, and only one positivity was found in CTRL samples stored at +4 °C and at +10 °C using the qualitative method. It can, therefore, be said that both cultures tested showed inhibitory activity against *L. monocytogenes*, with a slightly higher efficacy for *L. sunkii* (autochthonous) than *L. plantarum* (commercial).

The mean thermophilic lactic acid bacteria levels for all products (PSA, PSB, and CTRL) were 10^6^–10^7^ UFC/g at T_0_, and 10^7^–10^8^ at T_21_ and at T_90_; the counts of mesophilic lactic acid bacteria were 10^6^–10^8^ UFC/g at T_0_, and 10^6^–10^8^ UFC/g at T_21_, at T_90_, and at T_180_. For both mesophilic and thermophilic LAB, no significant differences (*p* > 0.05) in the three types (and the three batches) of experimental cheese were detected. These results are similar to those obtained in other challenge tests described in the literature [16,26,27,28]. The pH measurement showed that there were no substantial variations for any type of sample for the three batches analyzed. Throughout the storage period, the pH values never fell below 4.7. This feature, together with the detected a_w_ parameters, makes this product permissive to *L. monocytogenes* growth until the end of its shelf-life after 20 days of ripening.

This work presents an innovative protocol for utilizing bioprotective cultures. Traditionally, these cultures were applied to the product surface through spraying. However, this approach posed challenges for milk processing companies as it required an additional step in the cheesemaking process and specialized staff to perform it accurately. To address this issue and fulfill the requirements of dairy companies, this study proposes a protocol that introduces the inoculum directly into the boiler during the cheesemaking process. The results obtained from this work could significantly impact the activities of the food industry. In fact, the use of bioprotective LAB proved to be applicable to the production technology of Pecorino Sardo Dolce PDO and effective in improving the safety of RTE products in relation to contamination by *L. monocytogenes*. The results also indicate that samples inoculated with protective cultures did not show any presence of *L. monocytogenes* even after 180 days (T_180_). This test demonstrates that extending the shelf-life of these products to 180 days does not pose any issues in terms of the presence of this pathogen.

## 5. Conclusions

Pecorino Sardo Dolce PDO is produced using pasteurized sheep milk, following an industrial process that ensures a proper and fast acidification of the curd. During the ripening stage (ranging between 20 and –60 days), there is a low risk of contamination of the surface, but after ripening, when the cheese wheel is portioned, contamination of the cheese surface can occur. The physicochemical characteristics of Pecorino Sardo Dolce PDO with 20 days of ripening, considered in this study, place it in the category of RTE foods able to support the growth of *L. monocytogenes* [7]. A potential risk of *L. monocytogenes* growth on portioned cheese requires the adoption of appropriate preventive measures; in this frame, the use of LAB with antilisterial activity is intended as an additional procedure useful to ensure the safety of this product.

This is the first scientific research study that proposes the use of an indigenous strain of *Lactobacillus delbrueckii* subsp. *sunkii* as a bioprotective culture, and the inoculation of that culture into the milk before cheesemaking. Therefore, the data obtained are innovative and comparable only with studies on the use of other lactobacilli and commercial protective cultures. The choice of *L. sunkii* as a bioprotective culture was due to its growth optimum (45 °C) which made it suitable for inoculation in milk during cheesemaking, and its compatibility with the guidelines of production of Pecorino Sardo PDO cheese that stipulate that “the whole sheep’s milk is inoculated with bacteria from the area of origin”.

In conclusion, the results of the challenge test conducted in this study revealed the potential of bioprotective cultures, both autochthonous and commercial, to reduce the risk of *L. monocytogenes* contamination with a reduction in the level of pathogen contamination at the end of shelf-life.

Consumer safety is the most important objective of this study, and the use of bioprotective cultures in cheeses serves this purpose. The proposed technological innovation could provide food industry with further evidence on product safety while also offering guarantees of compliance with international legislation with a view to export. These results do not claim to guarantee the food safety of all PDO products, but the use of this method combined with good hygiene and manufacturing practices could be a winning strategy to avoid the problems of food contamination by foodborne pathogens.

## Figures and Tables

**Table 1:** LAB enumeration (Mean levels + standard deviation Log10 CFU/mL) evaluated in *scotta-innesto*.

Samples	LAB		
	Batch ^1^	Batch ^2^	Batch ^3^
*Scotta-innesto* PSA ^1^	7.7 ± 0.13	6.2 ± 0.1	7.6 ± 0.2
*Scotta-innesto* PSB ^2^	7.7 ± 0.3	6.6 ± 0.0	6.8 ± 0.1

^1^ *Scotta-innesto* produced by inoculating *Lactiplantibacillus plantarum* (Lyofast LPAL, Sacco System). ^2^ *Scotta-innesto* produced by inoculating *Lactobacillus delbrueckii* subsp. *sunkii*. ^3^ Mean of the plate count performed in triplicate ± standard deviation.

**Table 2:** Challenge test: results of physicochemical parameters. The pH and a_w_ data represent the mean measurements of three samples taken in triplicate from each of the three batches analyzed.

ID Samples and Time of Sampling		T° Storage ^1^	pH	a_w_
PSA ^3^ after breaking the curd		-	6.5 ± 0.058 ^a^	0.995 ± 0.001 ^a^
PSA after curd shrinking		-	5.4 ± 0.208 ^a^	0.992 ± 0.002 ^a^
PSB ^4^ after breaking the curd		-	6.5 ± 0.058 ^a^	0.997 ± 0.002 ^a^
PSB after curd shrinking		-	5.4 ± 0.058 ^a^	0.994 ± 0.001 ^a^
CTRL ^5^ after breaking the curd		-	6.5 ± 0.058 ^a^	0.996 ± 0.002 ^a^
CTRL after curd shrinking		-	5.5 ± 0.289 ^a^	0.993 ± 0.001 ^a^
PSA-blank ^2^	T_21_	+4 °C	4.9 ± 0.058 ^a^	0.982 ± 0.002 ^a^
PSA			5.0 ± 0.058 ^a^	0.988 ± 0.003 ^a^
PSB-blank			4.9 ± 0.058 ^a^	0.983 ± 0.001 ^a^
PSB			5.0 ± 0.508 ^a^	0.988 ± 0.006 ^a^
CTRL-blank			4.9 ± 0.058 ^a^	0.980 ± 0.007 ^a^
CTRL			4.9 ± 0.058 ^a^	0.988 ± 0.006 ^a^
PSA-blank	T_90_		4.9± 0.100 ^a^	0.971 ± 0.011 ^a^
PSA			4.9± 0.115 ^a^	0.973 ± 0.010 ^a^
PSB-blank			4.9± 0.058 ^a^	0.970 ± 0.007 ^a^
PSB			4.9 ± 0.100 ^a^	0.966 ± 0.015 ^a^
CTRL-blank			4.8 ± 0.115 ^b^	0.968 ± 0.013 ^a^
CTRL			4.8 ± 0.100 ^b^	0.969 ± 0.015 ^a^
PSA-blank	T_180_		5.0 ± 0.115 ^a^	0.963 ± 0.004 ^a^
PSA			5.0 ± 0.115 ^a^	0.968 ± 0.008 ^a^
PSB-blank			5.0 ± 0.153 ^a^	0.944 ± 0.041 ^a^
PSB			4.9 ± 0.058 ^a^	0.925 ± 0.042 ^a^
CTRL-blank			4.7 ± 0.100 ^a^	0.957 ± 0.023 ^a^
CTRL			4.7 ± 0.115 ^a^	0.958 ± 0.012 ^a^
PSA-blank	T_21_	+10 °C	4.9 ± 0.058 ^a^	0.975 ± 0.006 ^a^
PSA			5.0 ± 0.058 ^a^	0.982 ± 0.003 ^a^
PSB-blank			5.0 ± 0.058 ^a^	0.983 ± 0.001 ^a^
PSB			5.0 ± 0.115 ^a^	0.989 ± 0.002 ^a^
CTRL-blank			5.0 ± 0.100 ^a^	0.984 ± 0.001 ^a^
CTRL			4.9 ± 0.058 ^a^	0.987 ± 0.004 ^a^
PSA-blank	T_90_		4.9 ± 0.100 ^a^	0.962 ± 0.011 ^a^
PSA			4.9 ± 0.100 ^a^	0.967 ± 0.016 ^a^
PSB-blank			4.9 ± 0.058 ^a^	0.966 ± 0.009 ^a^
PSB			4.9 ± 0.058 ^a^	0.973 ± 0.008 ^a^
CTRL-blank			4.8 ± 0.115 ^b^	0.964 ± 0.007 ^a^
CTRL			4.8 ± 0.100 ^b^	0.976 ± 0.009 ^a^
PSA-blank	T_180_		5.1 ± 0.153 ^a^	0.948 ± 0.014 ^a^
PSA			5.0 ± 0.115 ^a^	0.951 ± 0.011 ^a^
PSB-blank			5.0 ± 0.058 ^a^	0.940 ± 0.023 ^a^
PSB			5.0 ± 0.058 ^a^	0.944 ± 0.020 ^a^
CTRL-blank			4.7 ± 0.058 ^a^	0.954 ± 0.013 ^a^
CTRL			4.8 ± 0.058 ^a^	0.952 ± 0.010 ^a^

^1^ T° storage: storage temperature of cheese during the challenge test (4 °C, to simulate correct storage conditions; 10 °C, to simulate thermal abuse). ^2^ blank: samples not experimentally contaminated with *L. monocytogenes*. They represent the control. ^3^ PSA: cheese with inoculum of *L. plantarum* bacteriocin producer (commercial protective culture, Lyofast LPAL, Sacco System). ^4^ PSB: cheese with inoculum of *Lactobacillus delbrueckii* subsp. *sunkii* (thermophilic autochthonous LAB isolated from Sardinian raw sheep’s milk) ^5^ CTRL: control production without inoculum of bioprotective cultures. Means with different letters were significantly different (*p* < 0.01).

**Table 3:** Challenge test: mesophilic and thermophilic LAB enumeration (mean levels + standard deviation Log10 CFU/mL) and qualitative and quantitative analysis of *L. monocytogenes* related to the three production batches. The data represent the mean measurements of three samples taken in triplicate from each of the three batches analyzed.

ID Samples and Time of Sampling		T° Storage ^1^	LAB 45 °C ^2^	LAB 30 °C ^3^	Lm QT ^4^	Lm QL ^5^
PSA ^7^ after breaking the curd		-	6.2 ± 0.252 ^a^	7.8 ± 0.436 ^a^	0 ± 0.0	-
PSA after curd shrinking		-	6.7 ± 0.100 ^a^	6.8 ± 0.100 ^a^	0 ± 0.0	-
PSB ^8^ after breaking the curd		-	6.5 ± 0.058 ^a^	7.5 ± 0.361 ^a^	0 ± 0.0	-
PSB after curd shrinking		-	6.7 ± 0.458 ^a^	6.8 ± 0.321 ^a^	0 ± 0.0	-
CTRL ^9^ after breaking the curd		-	5.8 ± 0.289 ^a^	7.5 ± 0.781 ^a^	0 ± 0.0	-
CTRL after curd shrinking		-	6.3 ± 0.656 ^a^	6.7 ± 0.379 ^a^	0 ± 0.0	-
PSA-blank ^6^	*T*_21_	+4 °C	8.0 ± 0.153 ^a^	7.8 ± 0.346 ^a^	0 ± 0.0	-
PSA			8.2 ± 0.351 ^a^	7.9 ± 0.404 ^a^	1.2 ± 1.0	4/6
PSB-blank			7.8 ± 0.115 ^a^	8.1 ± 0.058 ^a^	0 ± 0.0	-
PSB			8.0 ± 0.058 ^a^	8.1 ± 0.173 ^a^	1.6 ± 0.2	5/6
CTRL-blank			7.8 ± 0.473 ^a^	7.9 ± 0.321 ^a^	0 ± 0.0	-
CTRL			7.6 ± 0.300 ^a^	7.9 ± 0.153 ^a^	0.9 ± 0.8	5/6
PSA-blank	*T*_90_		7.7 ± 0.415 ^a^	7.9 ± 0.404 ^a^	0 ± 0.0	-
PSA			7.7 ± 0.252 ^a^	7.8 ± 0.173 ^a^	0 ± 0.0	3/6
PSB-blank			7.6 ± 0.321 ^a^	6.7 ± 1.290 ^a^	0 ± 0.0	-
PSB			7.5 ± 0.361 ^a^	7.0 ± 0.833 ^a^	0 ± 0.0	-
CTRL-blank			7.5 ± 0.346 ^a^	6.9 ± 0.700 ^a^	0 ± 0.0	-
CTRL			7.6 ± 0.115 ^a^	7.2 ± 0.569 ^a^	0 ± 0.0	4/6
PSA-blank	*T*_180_		7.4 ± 0.546 ^a^	7.8 ± 0.298 ^a^	0 ± 0.0	-
PSA			7.3 ± 0.351 ^a^	7.5 ± 0.285 ^a^	0 ± 0.0	-
PSB-blank			6.9 ± 0.719 ^a^	6.8 ± 0.638 ^a^	0 ± 0.0	-
PSB			6.7 ± 0.432 ^a^	6.8 ± 0.725 ^a^	0 ± 0.0	-
CTRL-blank			7.2 ± 0.067 ^a^	7.1 ± 0.281 ^a^	0 ± 0.0	-
CTRL			6.8 ± 0.155 ^a^	6.8 ± 0.341 ^a^	0 ± 0.0	1/6
PSA-blank	*T*_21_	+10 °C	8.1 ± 0.404 ^a^	7.8 ± 0.700 ^a^	0 ± 0.0	-
PSA			8.0 ± 0.231 ^a^	8.0 ± 0.500 ^a^	1.6 ± 0.2	5/6
PSB-blank			7.5 ± 0.252 ^a^	7.9 ± 0.153 ^a^	0 ± 0.0	-
PSB			8.0 ± 0.058 ^a^	8.1 ± 0.115 ^a^	0.6 ± 0.9	5/6
CTRL-blank			7.9 ± 0.361 ^a^	7.9 ± 0.153 ^a^	0 ± 0.0	-
CTRL			7.8 ± 0.115 ^a^	7.8 ± 0.208 ^a^	1.4 ± 0.3	5/6
PSA-blank	*T*_90_		8.0 ± 0.321 ^a^	8.0 ± 0.321 ^a^	0 ± 0.0	-
PSA			7.8 ± 0.058 ^a^	8.0 ± 0.153 ^a^	0 ± 0.0	2/6
PSB-blank			7.3 ± 0.361 ^a^	7.3 ± 0.458 ^a^	0 ± 0.0	-
PSB			7.6 ± 0.351 ^a^	7.4 ± 0.551 ^a^	0 ± 0.0	2/6
CTRL-blank			7.4 ± 0.058 ^a^	7.1 ± 0.681 ^a^	0 ± 0.0	-
CTRL			7.4 ± 0.153 ^a^	6.5 ± 0.721 ^a^	0 ± 0.0	3/6
PSA-blank	*T*_180_		6.9 ± 0.870 ^a^	7.4 ± 0.478 ^a^	0 ± 0.0	-
PSA			7.2 ± 0.164 ^a^	7.4 ± 0.035 ^a^	0 ± 0.0	-
PSB-blank			6.8 ± 0.429 ^a^	7.2 ± 0.123 ^a^	0 ± 0.0	-
PSB			7.0 ± 0.609 ^a^	7.3 ± 0.354 ^a^	0 ± 0.0	-
CTRL-blank			6.1 ± 0.576 ^a^	6.5 ± 0.263 ^a^	0 ± 0.0	-
CTRL			5.9 ± 0.188 ^a^	6.3 ± 0.370 ^a^	0 ± 0.0	1/6

^1^ T° storage: storage temperature of cheese during the challenge test (4 °C, to simulate correct storage conditions; 10 °C, to simulate thermal abuse conditions). ^2^ Lactic counts (45 °C): results of the enumeration of thermophilic lactic acid bacteria [20]. The data are expressed in Log10 UFC/g of the mean of all values with standard deviation in brackets. ^3^ Lactic counts (30 °C): the data are expressed in Log10 UFC/g of the mean ± standard deviation. ^4^ Lm QT: quantitative determination of *L. monocytogenes* [22]. The mean Log10 UFC/g of all data ± standard deviation. ^5^ Lm QL: qualitative determination of *L. monocytogenes* [21]. ^6^ blank: samples not experimentally contaminated with *L. monocytogenes*. They represent the control. ^7^ PSA: cheese with inoculum of *L. plantarum* bacteriocin producer (commercial protective culture, Lyofast LPAL, Sacco System). ^8^ PSB: cheese with inoculum of *Lactobacillus delbrueckii* subsp. *sunkii* (thermophilic autochthonous LAB isolated from Sardinian raw sheep’s milk). ^9^ CTRL: production control without inoculum of bioprotective cultures. Means with different letters were significantly different (*p* < 0.01).

## Data Availability

The data presented in this study are available within the article.
